# Atmospheric Hydroxyl Radical Reaction Rate Coefficient and Total Environmental Lifetime of α-Endosulfan

**DOI:** 10.1021/acs.est.3c06009

**Published:** 2023-10-13

**Authors:** Paulo C. Alarcon, Zoran Kitanovski, Mohsen Padervand, Ulrich Pöschl, Gerhard Lammel, Cornelius Zetzsch

**Affiliations:** † Multiphase Chemistry Department, Max Planck Institute for Chemistry, Mainz 55128, Germany; ‡ Department of Chemistry, Faculty of Science, University of Maragheh, Maragheh 55181-8311, Iran; § RECETOX, Faculty of Science, Masaryk University, Brno 60177, Czech Republic; ∥ Atmospheric Chemistry Research Unit, University of Bayreuth, Bayreuth 95447, Germany

**Keywords:** hydroxyl radical, reaction kinetics, organochlorine pesticide, persistent organic pollutant, multicompartmental distribution

## Abstract

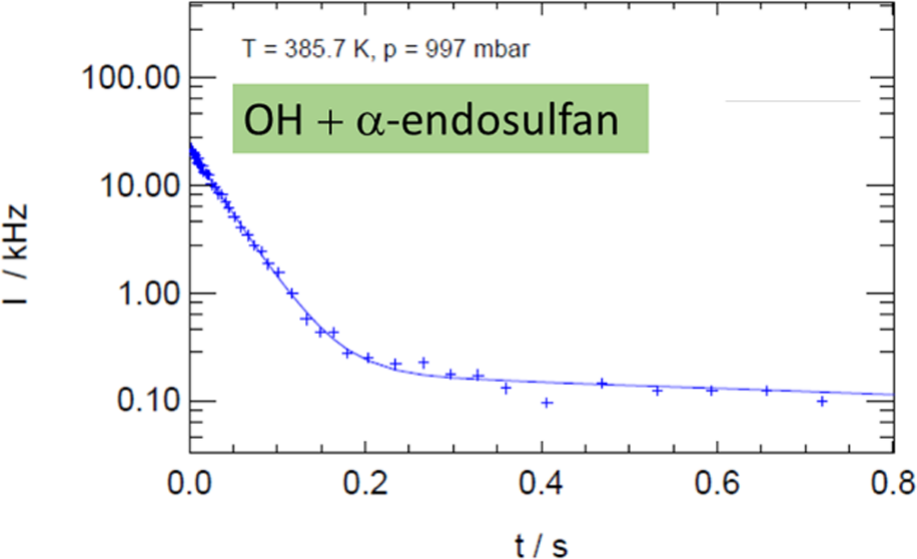

Endosulfan is a persistent organochlorine pesticide that was globally distributed before it was banned and continues to cycle in the Earth system. The chemical kinetics of the gas-phase reaction of α*-*endosulfan with the hydroxyl radical (OH) was studied by means of pulsed vacuum UV flash photolysis and time-resolved resonance fluorescence (FP-RF) as a function of temperature in the range of 348–395 K and led to a second-order rate coefficient *k*_OH_ = 5.8 × 10^–11^ exp(−1960*K*/*T*) cm^3^ s^–1^ with an uncertainty range of 7 × 10^–12^ exp(−1210*K*/*T*) to 4 × 10^–10^ exp(−2710*K*/*T*) cm^3^ s^–1^. This corresponds to an estimated photochemical atmospheric half-life in the range of 3–12 months, which is much longer than previously assumed (days to weeks). Comparing the atmospheric concentrations observed after the global ban of endosulfan with environmental multimedia model predictions, we find that photochemical degradation in the atmosphere is slower than the model-estimated biodegradation in soil or water and that the latter limits the total environmental lifetime of endosulfan. We conclude that the lifetimes typically assumed for soil and aquatic systems are likely underestimated and should be revisited, in particular, for temperate and warm climates.

## Abstract 2

The transformation of α-endosulfan by hydroxyl radicals in air is found to be much slower than previously assumed, suggesting that the lifetimes typically assumed for soil and aquatic systems are likely underestimated.

## 1 Introduction

Organochlorine pesticides (OCPs) are not readily degradable in soils, surface waters, and air and therefore have been distributed globally. Even after banning, i.e., without primary sources, OCPs continue to cycle in the Earth system, and their geographic and compartmental distributions are transient and shifting according to regionally varying compartmental lifetimes and climate change.^1,2^

The OCP endosulfan is very toxic for wildlife and humans.^3−9^ It had been produced since the 1950s, became one of the main OCPs used in history, was listed for elimination in the Stockholm Convention in 2011, and accordingly phased out in 2013. Endosulfan production reached up to about 20 kt/yr in 2010, and in total ca. 622 kt were produced.^10^ Largest amounts were applied and released in India, China, USA, Brazil, and Argentina, mostly as a substitute for the insecticide DDT that had been banned before. Application of the pesticide was via ground and aerial spraying, which is considered to correspond to high emission into the air.^11,12^ Endosulfan and its main degradation product, endosulfan sulfate, have been found distributed in air, surface waters, and soils in remote environments, far from source areas, including the open oceans and the Arctic.^13−22^

The substance applied in agriculture was technical endosulfan, which is a 7:3 mixture of α- and β-isomers (also called endosulfan I and II, respectively). The isomers have similar water solubilities, octanol–water partition coefficients, and vapor pressures over subcooled liquid.^5,6,23−26^ In the environment, β-endosulfan undergoes isomerization to α-endosulfan,^20,27^ which predominates^17^ and is preferentially volatilized from solid and aqueous surfaces.^28−32^

Endosulfan was listed as a persistent organic pollutant (POP) under the Stockholm Convention because of its long-range environmental transport potential and risk for adverse ecosystem and human health effects.^33^ As to persistence in the environment, half-times of weeks have been reported for the degradation of endosulfan in water and soil,^30,31,34−36^ which is significantly shorter than that for other organochlorine pesticides. In air, the photochemical lifetime of OCPs is generally dominated by the reaction of the gaseous molecule with OH radicals. The transformation by other reactive species, such as ozone and NO_3_ radicals and direct photolysis, are known to be inefficient for persistent OCPs in the gas phase,^37^ and experimental data are not available for α-endosulfan. Experimental data on rate coefficients for the reaction with OH radicals have not been reported for α-endosulfan in the literature so far. The AOPWIN model^38^ estimates an effective rate coefficient of *k*_OH_ = 8.17 × 10^–12^ cm^3^ s^–1^ (298 K), with a major contribution of H abstraction of 5.36 × 10^–12^ cm^3^ s^–1^ (from two tertiary and four secondary C–H bonds) and a contribution of OH addition to the olefinic double bond of 2.80 × 10^–12^ cm^3^ s^–1^, irrespective of the isomer. AOPWIN is based on structure–activity relationships at 298 K and does not account for steric hindrance or for possible stabilization of intermediates through intramolecular interactions. The error of the method is unknown. Steric hindrance is relevant for the bicyclic structure of the molecule, even more so for the addition of OH to its dichlorinated olefinic bond. A dossier prepared by the German Federal Environment Agency^5^ reported an earlier, more specific estimate of the inductive effect of the chlorine substituents and the sulfane group^39^ that delivered a value of *k*_OH_ = 1.8 × 10^–12^ cm^3^ s^–1^ (1.1 × 10^–12^ cm^3^ s^–1^ for addition and 0.7 × 10^–12^ cm^3^ s^–1^ for abstraction with an estimated error of an order of magnitude in both directions, neglecting steric hindrance as well). Furthermore, the dossier^5^ mentioned an experimental result obtained by the pulsed vacuum UV flash photolysis/resonance-fluorescence technique (FP-RF) in Ar at 130 mbar and 348 K with a rate coefficient of *k*_OH_ = (6.0 ± 1.5) × 10^–13^ cm^3^ s^–1^ for the α-isomer from a confidential, unpublished report to the manufacturer.^40^ In the study presented here, we used essentially the same apparatus to reinvestigate the OH + α-endosulfan reaction in He with an improved gas inlet system at higher temperature and pressure (348–395 K, 1 bar).

## 2 Experimental Methods

The instrument used has been described in detail elsewhere.^41,42^ The experiments involved time-resolved detection of OH radicals by resonance fluorescence (A^2^∑^+^ → X^2^Π) at λ= 308 nm. In summary, OH radicals were produced by pulsed vacuum UV flash photolysis of water vapor using a short arc xenon flash lamp (PerkinElmer Optoelectronics 1165 FX, Salem) as a photolytic light source at an energy of 540 mJ per flash. During the reaction, a He–H_2_O-α-endosulfan gas mixture flushed the reaction cell in which the photolysis of water vapor generates OH radicals. Another gas mixture of H_2_O and He was passed through a resonance lamp (mounted at right angles to the VUV photolysis beam and to the photomultiplier), where an electrodeless microwave discharge dissociated H_2_O to generate electronically excited hydroxyl (A^2^∑^+^) radicals. The fluorescence from the transition (A^2^∑^+^ → X^2^Π) leaving the lamp electronically excites the OH radicals in the reaction cell. After passing through a 308 nm interference filter, the resonance-fluorescence light from the reaction cell was focused onto the photocathode of a photomultiplier tube (Thorn-EMI, 9789QB, London, U.K.). The signal was processed using a photon-counting technique with a discriminator and was accumulated with a multichannel scaler board (EG&G Ortec, model ACE MCS, Oak Ridge), mostly during 4 s of observation time after each flash with a dwell time of 0.977 ms in each of the 4096 channels of the board (in a few experiments during 1 s each with a dwell time of 0.244 ms). The resonance-fluorescence signal was accumulated from a minimum of 40 flashes every 5 s, repeating the accumulation two times each, and the waiting time between each accumulation was at least 0.5 h. The experiments were automated by a personal computer, and the concentrations were controlled by feeding known flows of He through saturators with water at room temperature (291–301 K) and α-endosulfan powder at 351.5 K.

The gases used were He 99.9999% (Westfalen, Münster, Germany) and N_2_ 99.999% (Westfalen). Deionized water was used for the resonance lamp and as the photolytic precursor of OH in the gas saturation system. The vapor pressure of water was calculated using the equation: log(*P*_sat_/mbar) = 8.61 – 1948/(*T*_sat_/K – 24.15).^43^ The temperatures were determined with calibrated platinum resistance thermometers with an estimated accuracy of better than 0.5 K. The reactant, α-endosulfan >99% (Riedel-de Haen, Seelze, Germany), was used as received. Its vapor pressure was calculated from the equation log(*P*_sat_/mbar) = 12.128 – 5054.5*K*/*T*_sat_ (valid between 313 and 352 K) for α-endosulfan^44^ to be 5.60 × 10^–3^ mbar at 351.5 K. Figure S1 compares these recent vapor pressure data of the reactant determined by the vapor pressure balance technique with data measured by the manufacturer (similar technique^45^) and with measurements using the gas saturation system of the FP-RF, collecting the vapor in cooled dichloromethane at 240 K and analyzing by gas chromatography.^40^ The latter vapor pressure data are in better agreement with the new data, which are the basis for the present study.

This pesticide is a solid with a low vapor pressure. In order to avoid and minimize any loss by condensation and adsorption of α-endosulfan, the apparatus was modified by removing the needle valve between the gas saturation system and the resonance-fluorescence cell, connecting them by a glass line (heated to 370 K) directly. The much lower quenching of the resonance-fluorescence signal of OH by He in comparison with Ar enabled us to raise the total pressure to 1 bar and to employ higher levels of α-endosulfan than those at 130 mbar.

The initial OH radical concentration was estimated to be lower than 2 × 10^10^ cm^–3^ for the typical water concentration of 1.5 × 10^15^ cm^–3^ by comparison with an apparatus using the same kind of Xe flash lamp in a similar geometry.^46^ The total flow of He was kept constant at one standard liter per min. By adjusting the He flow through the saturator in 10 steps (typically between 0 and 5 standard cm^3^/min), the concentration of α-endosulfan was varied up- and downward between 10^12^ and 10^13^ cm^–3^, which is high enough to ensure that the reaction followed pseudo first-order kinetics. Furthermore, it is well below the extrapolated vapor pressure of about 1.5 × 10^–5^ mbar at 298 K (corresponding to 4 × 10^14^ cm^–3^).

## 3 Experimental Results and Discussion

The intensity of the resonance-fluorescence signal of OH was observed to decrease in a bi-exponential fashion after the flash according to the equation
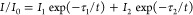
1and the rate coefficients, *k*_OH_, were determined from bi-exponential fits^47^ of the count rates (some examples are given in the Supporting Information, S2) by linear regressions of the initial decay rates, τ_1_^–1^, of the resonance-fluorescence signal versus the concentration of α-endosulfan according to the equation
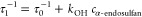
2where τ_0_^–1^ is the decay rate in the absence of the reactant (the so-called background reactivity). The second component of the bi-exponential decay was observed to be slower than 2 s^–1^ and to be unaffected by the level of α-endosulfan.

**Figure 1:**
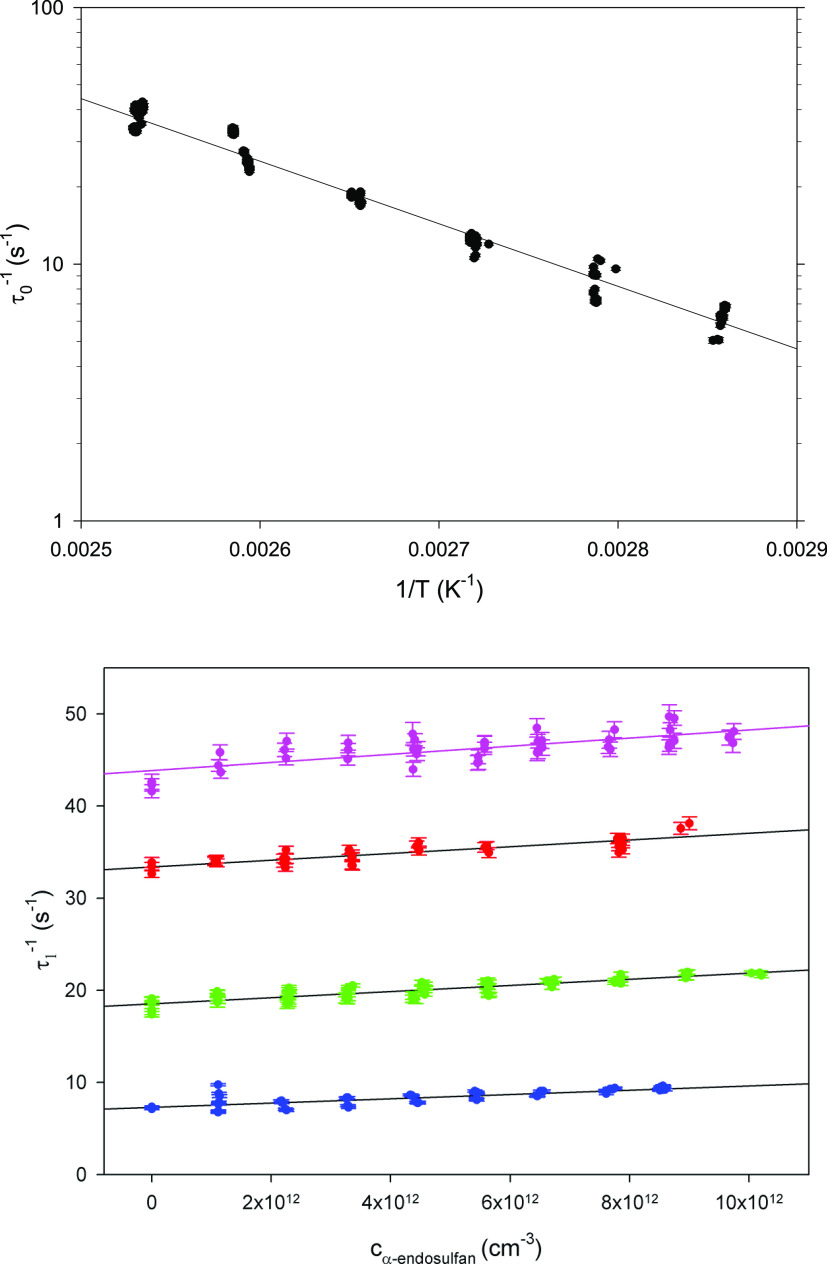
Decay rates of OH in the absence of α-endosulfan (a), corresponding to the Arrhenius expression τ_0_^–1^ = 5.5 × 10^7^ exp(−5610*K*/*T*) s^–1^ and (b) in the absence and presence of concentrations up to 10^13^ cm^–3^ and temperatures (from the bottom to top) of 359, 377, 387, and 395 K.

The low vapor pressure of α-endosulfan limits the range of decay rates and requires elevated temperatures for a significant increase of the decay rates of OH. On the other hand, the decay rates are observed to increase with temperature in the absence of reactant. As shown in Figure 1a, background reactivities,τ_0_^–1^, of the present study increased from 6 to 42 s^–1^ between 349 and 395 K, and decay rates in the presence of the reactant increased by less than 5 s^–1^, as shown in Figure 1b.

**Figure 2:**
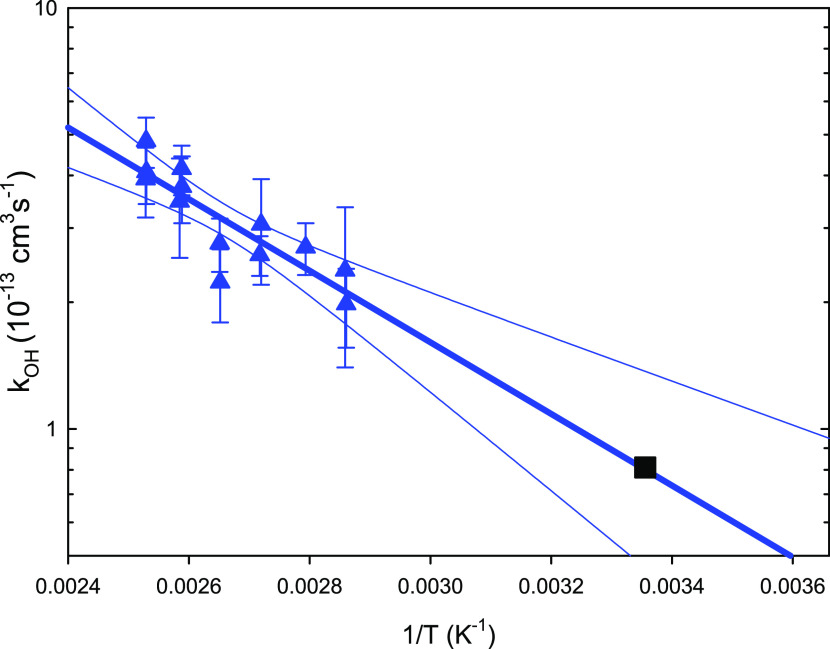
Arrhenius plot of the rate coefficients obtained for the reaction of OH with α-endosulfan in this study (blue triangles). The black square marks the extrapolation of the experimental data to 298 K, and 95% confidence limits are indicated; the model prediction of AOPWIN,^38^*k*_OH_ = 8.17 × 10^–12^ cm^3^ s^–1^ at 298 K, is higher by 2 orders of magnitude (not shown).

The rate coefficients experimentally determined in this study (after correcting for the background reactivity) are listed in SI, S3 (Table S1) and displayed in Figure 2. By least-squares fitting, we obtained the Arrhenius expression *k*_OH_ = 5.8 × 10^–11^ exp(−1960*K*/*T*) cm^3^ s^–1^ in He (with an uncertainty range from 7 × 10^–12^ exp (−1210*K*/*T*) to 4 × 10^–10^ exp(−2710*K*/*T*) cm^3^ s^–1^), delivering an extrapolated value (black square in Figure 2) of *k*_OH_ = 8.1 × 10^–14^ cm^3^ s^–1^ at 298 K and a corresponding atmospheric half-life *t*_1/2_ = 3.3 months (*t*_1/2_ = ln 2/(*k*_OH_*c*_OH_); assuming *c*_OH_ = 1 × 10^6^ cm^–3^). This rate coefficient is lower than reported from the previous study in Ar (*k*_OH_ = 6.0 × 10^–13^ cm^3^ s^–1^ at 348 K, corresponding to an atmospheric half-life of 2 weeks if assumed to be valid for 298 K^40^); and it is even 2 orders of magnitude lower than the structure–activity relationship-based model estimate of *k*_OH_ = 8.17 × 10^–12^ cm^3^ s^–1^ (AOPWIN^38^). The bath gas has normally no impact on abstraction reactions (the predominating mechanism for endosulfan, supported by the activation energy), and 1 bar of He should be sufficient^41,42^ for a complex molecule like endosulfan to bring any addition reaction close to the high-pressure limit similar to the 130 mbar of Ar in the previous study and to the collision efficiencies of N_2_ and O_2_. This holds even more so at room temperature, and we do not expect a significantly larger rate coefficient in the air.

Values of the observed background reactivities of the apparatus, τ_0_^–1^, were similar to those in previous experiments. These reflect the self-reaction of OH and reactions of OH with inherent trace impurities in the inert gas and from previous experiments, desorbing from surfaces and seals. The bi-exponential behavior of the OH signal might indicate a reversible addition of OH to the double bond, but it vanished and became almost imperceptible at higher temperatures. Since the bi-exponential behavior is observed in the absence of reactant as well, an evaluation for any reversible addition reaction of OH with α-endosulfan is not promising.

The data presented here of *k*_OH_ ± 2σ = (1.98 ± 0.43) × 10^–13^ cm^3^ s^–1^ at 349.6 K and (2.38 ± 1.0) × 10^–13^ cm^3^ s^–1^at 349.8 K (Table S1) in He at 1 bar are significantly lower than previous data in Ar at 130 mbar and 348 K,^40^ which had been obtained from two samples, one of them resublimated by the manufacturer and delivered rate coefficients (in units of 10^–13^ cm^3^ s^–1^) of 4.1 ± 0.8 (from a series of 48 decays) and 5.9 ± 1.8 (averaged from the last four series of 13 decays each, measured 7 days later from the resublimated sample). It had been recognized that the data did not provide the desirable long-term stability but decreased markedly over a time period of 2 weeks with the first sample and of 3 days of the last four series with the resublimated sample. This had been explained by volatile impurities, which were stripped by the Ar flow during the experiment and might take much longer than previously thought. However, a re-evaluation of the final series of the previous data shows a continued time trend with a halving time of 50 h that had been overlooked in the previous study. It appears that these values are compatible with the time trend of the previous data if the earlier measurements would have been continued for a few more days. The present data are obtained from a different, commercial sample (Riedel-de Haën, >99%) and did not show such a permanent decrease over time. This follows from the agreement of the rate coefficients (in units of 10^–13^ cm^3^ s^–1^) observed at 386.9 K (3.47 ± 0.94), at 386.3 K (3.76 ± 0.68), and at 386.4 K (4.15 ± 0.56), where the second and third measurements were taken 2 weeks later than the first one.

The rate coefficients of the present study lead to an extrapolated value of 8.1 × 10^–14^ cm^3^ s^–1^ at 298 K and correspond with annual mean photochemical half-lives of 3–12 months (288 K, 1 × 10^–6^ OH cm^–3^, *k*_OH_ uncertainty range) and 3–18 months in low to mid latitudes (i.e., 281–298 K, 1000 hPa, (0.3–1.2) × 10^6^ OH cm^–3 48^). Lower temperatures and Arctic levels of OH being a factor of 3–10 lower than in mid latitudes^48^ increase the half-life to more than 2 years. These half-lives are much longer than previously assumed (days to weeks). No experimental data exist for other possible photochemical reactions such as the ozone reaction of endosulfan or similar highly chlorinated compounds with an olefinic double bond nor for the reaction with the NO_3_ radical, which might decrease the half-life. In conclusion, the low value for *k*_OH_ underlines that the ban on this semivolatile persistent organic pollutant by the Stockholm Convention was justified and explains why long-range atmospheric transport to remote regions is efficient.

This experimental finding confirms that structure–activity relationship-based methods (2 orders of magnitude too high in this case) are not reliable for organic compounds beyond those compound classes actually used in the development of the estimation method.^37^

## 4 Consequences for Total Environmental Lifetime

For multicompartmental substances subject to diffusive surface-air mass exchange, the long-term trend in air (and in any other environmental compartment) following a ban (abrupt zero emission) should reflect the total environmental lifetime
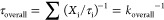
3with x_i_, τ_i_ = compartmental mass fractions and lifetimes, respectively, *k*_overall_ = total environmental degradation rate and total environmental half-life *t*_1/2_ = ln 2 × τ_overall_.^49^ This implies that surface reservoirs are not locked off and relaxation to equilibrium in the multimedia system is not retarded. We tested whether a correspondingly adjusted, expected total environmental half-life is in line with observations immediately following restrictions in various regions with observational data available, namely, Equatorial Africa,^50^ North American Gt. Lakes,^51^ and the Arctic^22^ (Supporting Information S4 with Table S2). The ban of endosulfan was effective globally by 2013, with very few exceptions.^52^ In the USA, it was effective 6 months earlier, with certain crops being exempted up to several years more, e.g., potato until 2015,^53,54^ although the corresponding amounts were small.^55^ For τ_i_, i = soil, sediment, and water (biodegradation and hydrolysis), we use literature values: Biodegradation in soils strongly depends on soil water content and temperature.^17,36^ In order to account for this uncertainty, calculations are done for both lower and upper estimates of *t*_1/2 soil_, i.e., 7 and 75 days at 298 K.^17^ For degradation in water, we adopt a rate determined for nonsterile seawater (3.65 × 10^–7^ s^–1^ at 294 K^35^), which should reflect the combination of biodegradation and hydrolysis. Recently, more rapid hydrolysis at seawater pH and temperature, corresponding to *t*_1/2_ = 2–5 days, was suggested, however, based on measurements in deionized water.^56^ For degradation in unsterile sediment, 3.65 × 10^–7^ s^–1^ at 298 K was derived.^35^ We assume a default slope for temperature dependencies of biodegradation, i.e., doubling per 10 K for *k*_soil_, *k*_sediment_, and *k*_water._(17,56,57) τ_water_ is particularly uncertain for seawater. For τ_air_, our *k*_OH_ result, accounting for lower and upper reactivity limits (i.e., *k*_OH_(*T*) = 4 × 10^–10^ exp(−2710*K*/*T*) and 7 × 10^–12^ exp(−1210*K*/*T*) cm^3^ s^–1^, respectively) and regional annual mean *c*_OH_(48) are used. Note that hereby we neglect the possible contribution of other atmospheric removal processes, such as dry and wet deposition or degradation by other oxidants, which may constitute an overestimation of τ_air_. Dry and wet deposition could be limiting with partitioning of endosulfan to the particulate phase, negligible in most observations, and possibly relevant only for high altitudes and high latitudes.

**Figure 3:**
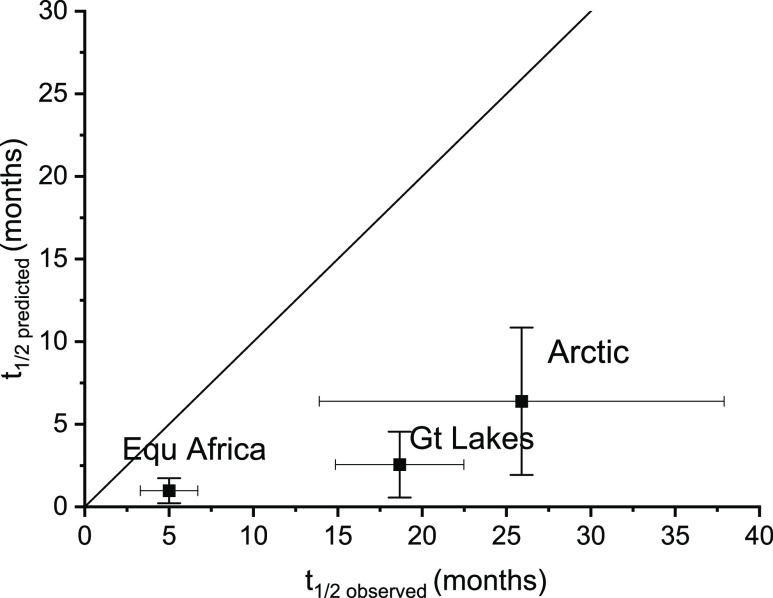
Predicted (multimedia model) and observed half-lives. Error bars of predicted values reflect a range of *k*_overall_ regressed over 3 years at 1 (Great Lakes) or 2 (Equatorial Africa, Arctic) sites, and uncertainties of *k*_OH_ (temperature dependence) and *k*_soil_.

The compartmental mass fractions *x*_i_ are taken from the output of a multimedia model under steady-state condition^58^ (lvl III, v2.80). The model uses default domain dimensions and regionally explicit temperature and surface distributions, i.e., land and water area fractions, and calculates the compartmental distribution of endosulfan under continuous emissions advected in the air into the model domain. More details are given in SI, S4. The so-predicted total environmental half-lives *t*_1/2_ are shown in Figure 3. They underestimate the observations by a factor of 2–8.

Predicted values of *t*_1/2_ are longer by 16, 0.4, and 0.04% for Equatorial Africa, the North American Great Lakes region and the Arctic, respectively, than would have been the case if the uncorrected, previous value for *k*_OH_ had been used (similar temperature dependence) and are longer by a factor of 3 for Equatorial Africa and by 6 and 0.6% for the North American Great Lakes region and the Arctic, respectively, than the model would have predicted if the AOPWIN^38^*k*_OH_ had been used. These differences are calculated assuming the same temperature dependence for the rate, namely, the one measured in this study. The effect of a correction of *k*_air_ on *k*_overall_ and total environmental half-life is strongest for the region with the highest temperature and, hence, the highest mass fraction in the air, i.e., Equatorial Africa (*x*_air_ = 3–23% but <1% in the other regions). The underestimation of the half-lives of the pollutant in various climates indicates that compartmental lifetimes τ_i_ other than τ_air_ are underestimated, in particular, in temperate and warm climates, or the relaxation to equilibrium in the multimedia system may be retarded and steady-state conditions may not be achieved. However, the unavailability of endosulfan in soils for diffusive air-surface mass exchange appears unlikely, considering that the reservoir is concentrated in the top soils.^59,60^ The conclusion on lifetimes τ_i_ other than τ_air_ is robust, because the value for τ_air_ is an upper estimate, neglecting other atmospheric sink processes. Underestimation of half-life in seawater and soil as the source of the discrepancy is supported by observations of endosulfan levels in open ocean waters (Bering Sea and Arctic Ocean^61^) and in soils far from application (Canadian Rocky Mtns.^62^), suggesting resistance to rapid degradation. Specification of biodegradation rates is key for further constraining the persistence of endosulfan and the pollutant’s fate in the Earth system.

## Supporting Information Available

Arrhenius plot of vapor pressure data of a-endosulfan; examples of bieponential fits to resonance-fluorescence signals and residuals; table of rate coefficients, *k*_OH_, and standard deviations; description of total environmental lifetime and table of observed half-lives in air and predicted total environmental half-life of α-endosulfan during years following ban for various regions (PDF)
