# Supplementary material for: Atmospheric Hydroxyl Radical Reaction Rate Coefficient and Total Environmental Lifetime of α-Endosulfan
Source: Environ Sci Technol. 2023 Oct 13;57(42):15999–6005. doi: 10.1021/acs.est.3c06009 (PMC10603777; doi:10.1021/acs.est.3c06009)
Supplement: Supplementary file 1 — es3c06009_si_001.pdf [file es3c06009_si_001.pdf]

Supporting Information

Atmospheric Hydroxyl Radical Reaction Rate Coefficient and Total Environmental Lifetime of α-Endosulfan

Paulo C. Alarcon[1], Zoran Kitanovski[1], Mohsen Padervand[1,2], Ulrich Pöschl[1], Gerhard Lammel[1,3], Cornelius Zetzsch[1,4*]

[1]Multiphase Chemistry Department, Max Planck Institute for Chemistry, Mainz, Germany
[2]Department of Chemistry, University of Maragheh, Maragheh, Iran
[3]RECETOX, Faculty of Science, Masaryk University, Brno, Czech Republic
[4]Atmospheric Chemistry Research Unit, University of Bayreuth, Bayreuth, Germany

Content: 5 pages, 2 tables and 2 figures

S1 Vapor pressure

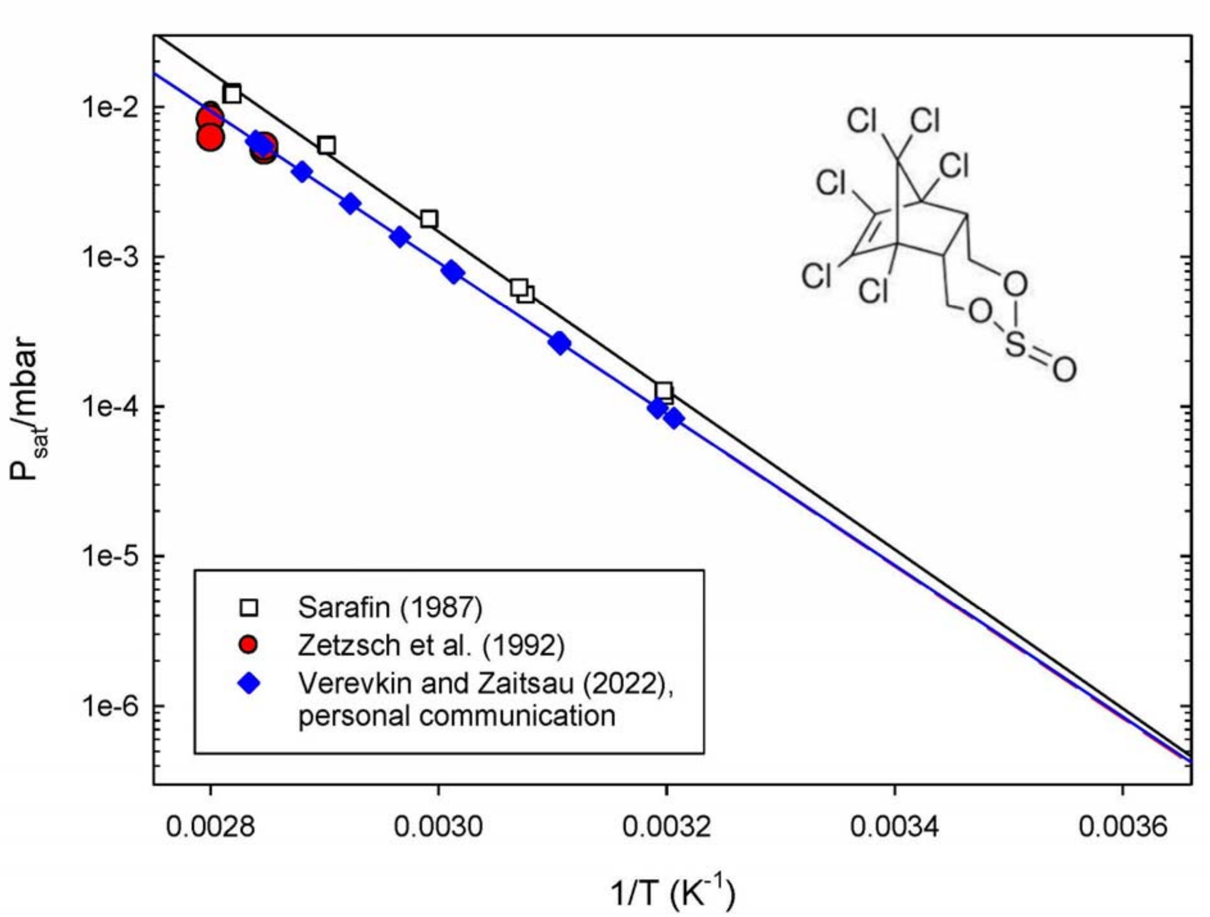

Fig. S1: Arrhenius plot of the vapor pressure data of α-endosulfan obtained by the gas balance with Knudsen effusion[1-2] and by the gas saturation technique[3].

S2 Biexponential fits

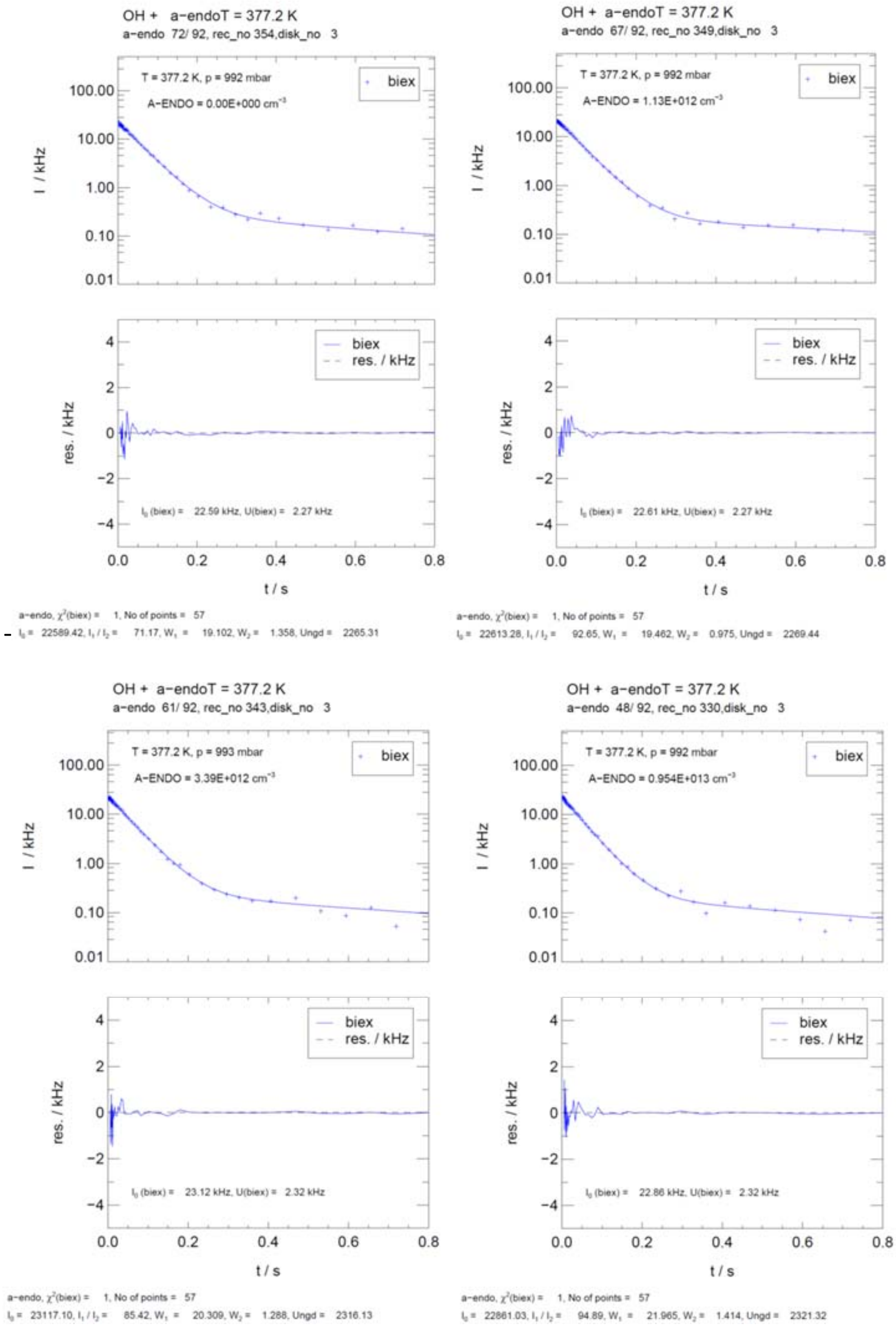

Fig. S2: Examples of biexponential fits to resonance fluorescence signals and residuals at various levels of α-endosulfan

S3 Rate coefficients

Table S1: Rate coefficients, k_{OH}, and standard deviations, σ, in units of 10^{-13} cm^3 s^{-1}.

T/K	k_{OH}	σ
349.6	1.98	0.21
349.8	2.38	0.49
358.0	2.70	0.19
367.7	3.06	0.43
368.0	2.59	0.14
377.1	2.23	0.22
377.2	2.76	0.20
386.9	3.47	0.46
386.3	3.76	0.34
386.4	4.15	0.28
395.3	4.08	0.33
395.4	4.83	0.33
395.5	3.92	0.37

S4 Total environmental lifetime

Compartmental distributions: Apart from site-unspecific settings, the steady state (level III [4]) multimedia model predicts the multicompartmental mass distribution as determined by the land-sea distribution (surface area fraction), temperature, and compartmental degradation rates. The latter are input temperature dependent (using the here determined temperature dependence for air and a default temperature dependence for all other compartments; see above [5]) and as lower and upper estimates for air and soil (using the here determined uncertainty of k_{OH} and the reported uncertainty range of $t_{1/2\ soil}$ i.e., 7-75 days at 298 K; Weber et al., 2010) and fixed values for other compartments. These input parameters in 3 different climates result in endosulfan partitioning to soil by 47-96%, to water by 0.7-30%, to sediment by 0.14-23% and to air by 0.01-23%. Note that most of the range for air is not reflecting difference in temperature, but the range in soil degradation rate.

Selection of observational data sets: Concentration of endosulfan in air, similar to all semivolatile OCPs in the absence of (or with only negligible) primary emissions, shows a pronounced seasonality i.e., summer maximum. Therefore, exponential regressions to decline of concentration after ban 2012/13 need to address complete annual cycles, minimum two. Furthermore, the data are suitable, if remaining distant from limit of quantification and, if the time series was apparently dominated by the seasonality and decline after ban, whereas other features, eventually indicating regional primary emissions after ban were negligible. These

criteria apply for time series of concentration of endosulfan in air from Villum Research Station, Pallas [6] (retrieved from [7]), Chicago [8] (retrieved from [9]), Abetefi and Mt. Kenya [10] (retrieved from [11]).

Table S2: Observed half-lives in air and predicted total environmental half-life of *α*-endosulfan during years following ban for various regions

Region	Climate parameters		Predicted $t_{1/2}$ (mo) [b]	Observed $t_{1/2}$ (mo) site
	air and soil temperature, water and sediment temperature	c_{OH} [a], area fraction of water		
Arctic	273 K, 272 K [c]	$(0.25\pm0.15)\times10^6$ cm^{-3}, 0.65	6.4±4.5	13±1 Pallas (Finland, 67°N), 37±2 Villum (Greenland, 82°N) [d]
North American Gt. Lakes	283 K, 283 K	0.67×10^6 cm^{-3}, 0.50	2.6±2.0	19±4 Chicago (USA, 42°N) [e]
Equatorial Africa	298 K, 298 K	1.38×10^6 cm^{-3}, 0.05	0.97±0.76	5.0±1.7 Abetefi (Ghana, 6°N), 3.7±0.4 Mt. Kenya (Kenya, 0°N) [f]

[a] annual mean at respective latitude and pressure level [12]
[b] range reflects uncertainty of $t_{1/2\ soil}$ = 41±34 d [13]
[c] annual mean [14-15]
[d] exponential regression to time series of 2 years from July 2012 in Pallas and 3 years from January 2013 in Villum Research Station [6-7]
[e] exponential regression to time series of 2 or 3 years from July 2012 [8-9]
[f] exponential regression to time series of 3 years from January 2013 [10-11]

Supplementary references

(1) Sarafin, R., 1987. *Hoe 002671 (endosulfan), Hoe 052618 (α-endosulfan) and 052619 (β-endosulfan) – Vapour pressures*, Report A 36734, (B) 153/87, Hoechst AG, Angewandte Physik/Analytisches Laboratorium, Frankfurt/M., Germany.

(2) Verevkin, S.P.; Zaitsau, D.H., 2022. University of Rostock, Rostock, Germany, Personal Communication.

(3) Zetzsch, C.; Elend, M.; Knispel, R.; Koch, R.; Siese, M., *Photochemisch-oxidativer Abbau von α-Endosulfan durch OH-Radikale.* Unpublished report to Hoechst AG by Fraunhofer-Institut für Toxikologie und Aerosolforschung, Hannover, AgrEvo Doc. No. A48146, Frankfurt/M., 1992.

(4) Mackay, D., 2001. *Multimedia Environmental models – the Fugacity Approach*, 2nd ed., CRC Press, Boca Raton, USA, 272 pp.

(5) Technical Guidance Document in Support of The Commissions Directive 93/67/EEC on Risk Assessment for the Notified Substances and the Commission Regulation (EC) 1488/94 on Risk Assessment for Existing Substances, European Chemicals Bureau, Ispra, Italy, 1996.

(6) Wong, F.; Hung, H.; Dryfhout-Clark, D.; Aas, W.; Bohlin-Nizzetto, P.; Breivik, K.; Nerentorp Mastromonaco, M.; Brorström-Lundén, E.; Olafsdottír, K.; Sigurdsson, A.; Vorkamp, K.; Bossi, R.; Skov, H.; Hakola, H.; Barresi, E.; Sverko, E.; Fellin, P.; Li, H.; Vlasenko, A.; Zapevalov, M.; Samsonov, D.; Wilson, S., Time Trends of Persistent Organic Pollutants (POPs) and Chemicals of Emerging Arctic Concern (CEAC) in Arctic Air from 25 years of Monitoring. *Sci. Total Environ.* **2021**, 775, 145109.

(7) EMEP, Co-operative programme for monitoring and evaluation of the long-range transmission of air pollutants in Europe (EMEP), Atmospheric measurement data base EBAS, Norwegian Institute for Air Research (NILU). URL: https://ebas.nilu.no (accessed 2023-04-30).

(8) Hites, R.A., Statistical Approach for Assessing the Stockholm Convention's Effectiveness: Great Lakes Atmospheric Data, *Environ. Sci. Technol.* **2019**, 53, 8585–8590.

(9) IADN, 2022. Environmental Data Base. Data Visualization, Indiana University. URL: https://iadnviz.iu.edu/datasets/index.html (accessed 2022-09-11).

(10) White, K.B.; Kalina, J.; Scheringer, M..; Přibylová, P.; Kukučka, P.; Kohoutek, J.; Prokeš, R.; Klánová, J., Temporal Trends of Persistent Organic Pollutants Across Africa after a Decade of MONET Passive Air Sampling. *Environ. Sci. Technol.* **2021**, 55, 9413-9424.

(11) GENASIS, 2022. Global Environmental Assessment Information System, environmental data repository, URL: https://www.genasis.cz/index-en.php?pg=data-sources.

(12) Spivakovsky, C.M.; Logan, J.A.; Montzka, S.A.; Balkanski, Y.L.; Foreman-Fowler, M.; Jones, D.B.A.; Horowitz, L.W.; Fusco, A.C.; Brenninkmeijer, C.A.M.; Prather, M.J.; Wofsy, S.C.; McElroy, M.B. Three-Dimensional Climatological Distribution of Tropospheric OH: Update and Evaluation *J. Geophys. Res.* **2000**, 105, 8931.

(13) Weber, J.; Crispin, J.; Halsall, Muir, D.; Teixeira, C.; Small, J.; Solomon, K.; Hermanson, M.; Hung, H.; Bidleman, T. Endosulfan, a Global Pesticide: A Review of its Fate in the Environment and Occurrence in the Arctic, *Sci. Total Environ.* **2010**, 408, 2966–2984.

(14) Krampe, D.; Kauker, F.; Dumont, M.; Herber, A., Snow and meteorological conditions at Villum Research Station, Northeast Greenland: on the adequacy of using atmospheric reanalysis for detailed snow simulations, *Front. Earth Sci. Sec. Cryospheric Sci.* **2023**, 11, 1053918

(15) Weather and Climate, *The Global Historical Weather and Climate Data*, URL: https://tcktcktck.org/finland/lapland (accessed 2023-06-25)
